# Effective Diagnosis of Prostate Cancer Based on mRNAs From Urinary Exosomes

**DOI:** 10.3389/fmed.2022.736110

**Published:** 2022-03-23

**Authors:** Jiahua Gan, Xing Zeng, Xiong Wang, Ya Wu, Ping Lei, Zhihua Wang, Chunguang Yang, Zhiquan Hu

**Affiliations:** ^1^Department of Urology, Tongji Hospital, Tongji Medical College, Huazhong University of Science and Technology, Wuhan, China; ^2^Department of Laboratory Medicine, Tongji Hospital, Tongji Medical College, Huazhong University of Science and Technology, Wuhan, China; ^3^Wuhan YZY Medical Science and Technology Co., Ltd., Wuhan, China; ^4^Department of Immunology, Tongji Medical College, Huazhong University of Science and Technology, Wuhan, China

**Keywords:** prostate cancer, diagnosis, prognosis, biomarker, urinary exosome, PSA

## Abstract

**Background:**

Novel non-invasive biomarkers are urgently required to improve the diagnostic sensitivity and specificity of prostate cancer (PCa). Therefore, the diagnostic value of following candidate genes (ERG, PCA3, ARV7, PSMA, CK19, and EpCAM) were estimated by testing mRNAs from urinary exosomes of patients with primary PCa.

**Methods:**

Exosomes were obtained using size-exclusion chromatography (SEC), out of which RNAs were extracted, then analyzed by quantitative reverse transcription-polymerase chain reaction according to manufacturer’s protocol.

**Results:**

The expression of urinary exosomal ERG, PCA3, PSMA, CK19, and EpCAM were significantly increased in patients with PCa compared with healthy males. In addition, the levels of urinary exosomal ERG, ARV7, and PSMA were intimately correlated with the Gleason score in PCa patients (*P* < 0.05). The receiver operating characteristic curves (ROCs) showed that urinary exosomal ERG, PCA3, PSMA, CK19, and EpCAM were able to distinguish patients with PCa from healthy individuals with the area under the curve (AUC) of 0.782, 0.783, 0.772, 0.731, and 0.739, respectively. Urinary exosomal PCA3 and PSMA distinguished PCa patients from healthy individuals with an AUC of 0.870. Combination of urinary exosomal PCA3, PSMA with serum PSA and PI-RADS achieved higher AUC compared with PSA alone (0.914 and 0.846, respectively). Kaplan-Meier curves demonstrated that PCA3, ARV7, and EpCAM were associated in androgen-deprivation therapy (ADT) failure time which is defined as from the initiation of ADT in hormone-sensitive stage to the development of castration-resistant prostate cancer.

**Conclusion:**

These findings suggested that mRNAs from urinary exosomes have the potential in serving as novel and non-invasive indicators for PCa diagnosis and prediction.

## Introduction

Prostate cancer (PCa), as one of the most common cancers and the leading cause of cancer-related death in males, mainly occurs to men over 50 years old (1). Early detection strategy of PCa is primarily accomplished by evaluating the expression level of prostate-specific antigen (PSA) in the blood and MRI. Unfortunately, because of the limited sensitivity and specificity of PSA detection, benign prostatic hyperplasia (BPH) or prostatitis can’t be reliably distinguished from clinically significant PCa (2). Therefore, the development of new accurate and non-invasive biomarkers has been urgently needed for the diagnosis of PCa at the early stage.

Recently, the field of extracellular vesicles (EVs) has been rapidly developing. EVs, secreted by prokaryotic and eukaryotic cells, play important roles in intercellular communication by delivering proteins, nucleic acids, and enzymes to target cells. Exosomes (30–200 nm) belong to one kind of the EVs, which are formed by multivesicular bodies (MVBs) fusing into plasma membrane of the cell. In particular, the loads carried by exosomes include specific components of proteins, RNA, lipids, and DNA, which function as mediums to transmit information to target cells and induce shifts in genetic and epigenetic regulations (3). Because of the lipid bilayer structure, urinary exosomes possess stability in resisting external interference. Accumulative researches have indicated that the relative abundance of these urinary exosomes is altered in kinds of diseases of kidney, bladder, and prostate (4–7). Thus, it has been hypothesized that some of these exosome-derived molecules may have the potential of being applied as biomarkers with high sensitivity in the corresponding diagnosis.

Numerous studies have reported the changes in the relative RNAs abundance of ERG, PSMA, ARV7, PCA3, CK19 and EpCAM in PCa tissues (8–10). For example, previous studies have reported the detection of fusion between TMPRSS2 and ERG in prostate cancer tissues, and high expression of PSMA in advanced PC or mCRPC tissues which is related to related to higher tumor stage/Gleason score/preoperative PSA levels (11, 12). PCA3, one of the most commonly used urine markers for the prognosis of PCa, has been reported to have a sensitivity of 65%, specificity of 73% in detecting prostate cancer, respectively (13, 14), while AR-V7 positive was detected in a large portion of CRPC. Other researchers have proved higher EpCAM expression level in PCa samples than in benign and normal samples by immunohistochemistry (15). However, these findings have hinted us with the potential significance of these biomarkers in the diagnosis of PCa, however, they did not focus on simplify the access and detection method of samples or how to promote sensitivity and precision since these markers did no show extraordinary advantages compared with present clinical markers. Hence, standing on the giant’s shoulder, we stepped on the field of the combined-detection of urinary exosome-derived mRNAs of these markers. By this mean, we actually gained ideal results which have prospect in future clinical application. Urine holds the opportunity to replace plasma and be a promising and challenging substitute source of a new biomarker in PCa. In the past years, some researchers have identified the urinary RNA profiles of PCA (16). However, the evaluation of indicators based on different RNA combination has not been conducted or reported yet. No joint RNA urinary exosomes which can be utilized for early detection has been examined. More in-depth explorations are needed to further examine the potential of RNA in the diagnosis of PCa. Therefore, based on the previous reports, in our study, we aimed to figure out the potential of six urinary-exosome-originated cancer-related RNAs (ERG, PSMA, ARA7, PCA3, CK19, and EpCAM) in being biomarkers for the detection and prognosis of PCa.

## Materials and Methods

### Patients and Samples

Urine samples were obtained from January 2019 to June 2019 in Tongji Hospital, Huazhong University of Science and Technology with informed consents. A total of 63 PCa patients and 61 healthy individuals were enrolled in the validation cohort. Inclusion criteria were set as: patients who undergone prostate needle biopsy and were diagnosed of PCa by histology detection. They were not previously treated with neoadjuvant androgen deprivation therapy and had no prior history of malignancy. The control group was defined as: males with total PSA < 4 ng/mL; aged between 45 and 80 years old; no significant change was observed by ultrasound imaging;no prostatitis. The first morning urine samples (30 ml) of all participants were collected without any operation of transrectal prostate examination and massage. This study was approved by the Ethics Commission of Tongji Hospital, Tongji Medical College, Huazhong University of Science and Technology (TJ-IRB20190957). Urine samples were tested directly right after collection, then centrifuged at 10,000 × *g* for 20 min to remove cellular debris and deposits. The supernatant (30 ml) was filtered by a 0.22 μm pore size filter membrane in 3 min. Then the filtrate was concentrated to a volume of 300 μL by an Amicon Ultra-15-ml centrifugal filtration device (100 kDa) according to the protocol. PBS was added into the ultra-centrifugal filter unit to prepare for the following centrifugation at 6000*g* for 15 min, which was repeated for three times. After buffer replacement, the final solution was kept in 1.5 ml EP tubes and stored at −80°C.

### Exosomes Isolation

The excretion column (Exosupur, Beijing, China) was applied according to the protocol. The excretion column was equilibrated at room temperature, kept vertical in the excretion rack, and rinsed with three times volume of PBS. After rinsing, the bottom cap was sealed, and 1–2 ml of PBS was added inside. Then the bottom cap of the excretion column was removed, and the PBS on top of the upper sieve plate was aspirated and discarded. Finally, 500 μL of the fraction was immediately collected after the sample was added into the column, during which the filtered PBS was used as elution buffer.

### mRNA Isolation

mRNAs were extracted from exosomes using an exoRNeasy Midi Kit (No. 77144, Qiagen, German) according to the manufacturer’s protocol and stored at −80°C.

### Quantitative Reverse Transcription-Polymerase Chain Reaction

The concentration of RNA were quantified by NanoDrop 2000 spectrophotometer (Thermo Fisher Scientific, United States). The cDNA was obtained from total RNA via the Quantitect Reverse Transcription Kit (Qiagen, Hilden, Germany) according to the protocol. The cDNA generated from total RNA was utilized for reverse transcription reactions by One Step PrimeScript RT-PCR (Takara). The expression levels of ERG, PSMA, ARV7, PCA3, CK19, and EpCAM were measured by the same kit. qRT-PCR was performed in a LightCycler480 real-time PCR system (Roche, Hoffmann-La Roche Ltd., Basel, Switzerland). Primers for mRNAs were purchased from Takara. The primer sequences were as follows: ERG:F:5′-GCGTCCTCAGTTAGA TCCTTATCAG-3′, P:5′-ACAAGTAGCCGCCTTG-3′, R:5′-CT GGCCACTGCCTGGATT-3′; PSMA:F:5′-CGCAGTAGAGCAG CAGCACA-3′, P:5′-CTCCTTCACGAAACCGA-3′, R:5′-AACC ACCCGAAGAGGAAGC-3′; PCA3: F:5′-CTCGCATTTGTGGG TTCT-3′, P:5′-CTTGCATTAGGTCTCAGCT-3′, R:5′-CTCC AAACCTGGTAAATGATTCCT-3′; CK19:F:5′-AGAAGAACCA TGAGGAGGAAATCA-3′, P:5′-TCAGTGTGGAGGTGGATT-3′, R:5′-ATCTTGGCGAGATCGGTGCC-3′; EpCAM:F:5′-TC TAAGAAAATGGACCTGAC-3′, P:5′-TAAATGGGGAACAAC TGGAT-3′, R:5′-ATCTTGGCGAGATCGGTGCC-3′. RT-qPCR reaction was conducted with following parameters: 95°C for 5 min, 95°C for 15 s and 60°C for 35 s and processed for 45 cycles. The relative amount of each mRNA was calculated by following equation: ΔCt = Ct mRNA −Ct B2M. ΔCt method was also utilized in Comparative quantification.

### Statistical Analyses

Statistical analysis was conducted by SPSS version 22.0. Receiver operating characteristic (ROC) curve analysis with 95% confidence intervals (95% CIs) was conducted to assess the sensitivity and specificity of different biomarkers by calculating the area under the curve (AUC). Student’s *t*-test was applied for statistical comparison of categorical variables. In PCa patients, the same tests were conducted to calculate the significance of differences in marker values with Gleason score. Logistic regression analysis was performed to double confirm the accuracy of the analysis results. The differences were considered statistically significant when *P* value < 0.05. The relationship between exosomal RNA levels and time to ADT failure was assessed using Kaplan-Meiers survival curves.

## Results

Patients’ characteristics demographic and clinical data were displayed in Table I, which included PSA serum levels, biopsy Gleason score, PI-RADS, and clinical stage. Urinary exosomes were obtained from 63 of these patients (Table 1).

**TABLE 1:** Demographic and clinicopathological characteristics of the patients included in the study.

Parameters	PCa	Healthy subjects groups
Number	63	61
**Age (years)**		
Median	72	60
Range	60–86	45–79
**PSA serum levels (ng/ml)**		
Median	20.7	1.09
Range	8.3–184	0.12–3.15
**Biopsy Gleason score**		
≤7	34	–
≥8	29	–
**Clinical stage**		
T1	8	–
T2-T3	32	–
T4	23	–
**PI-RADS**		
2	2	–
3	9	–
4	10	–
5	42	–

**FIGURE 1:**
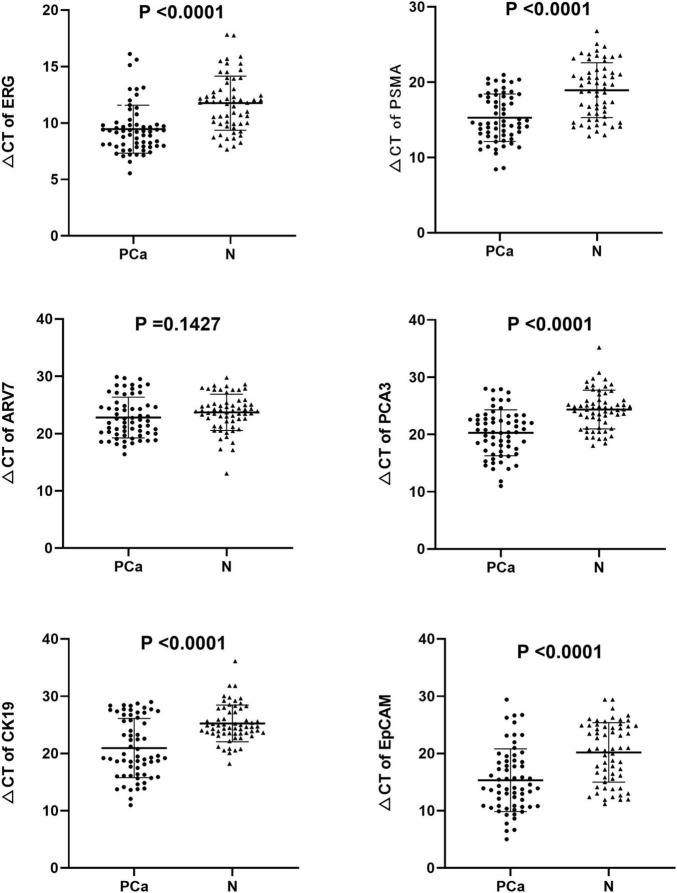
Expression levels of the six mRNAs in PCa patients (*n* = 63) and healthy subjects groups (*n* = 61) by qRT-PCR in urinary exosomes. Statistical test: Student’s *t*-test. P < 0.05 was considered statistically significant (Difference between two groups).

**FIGURE 2:**
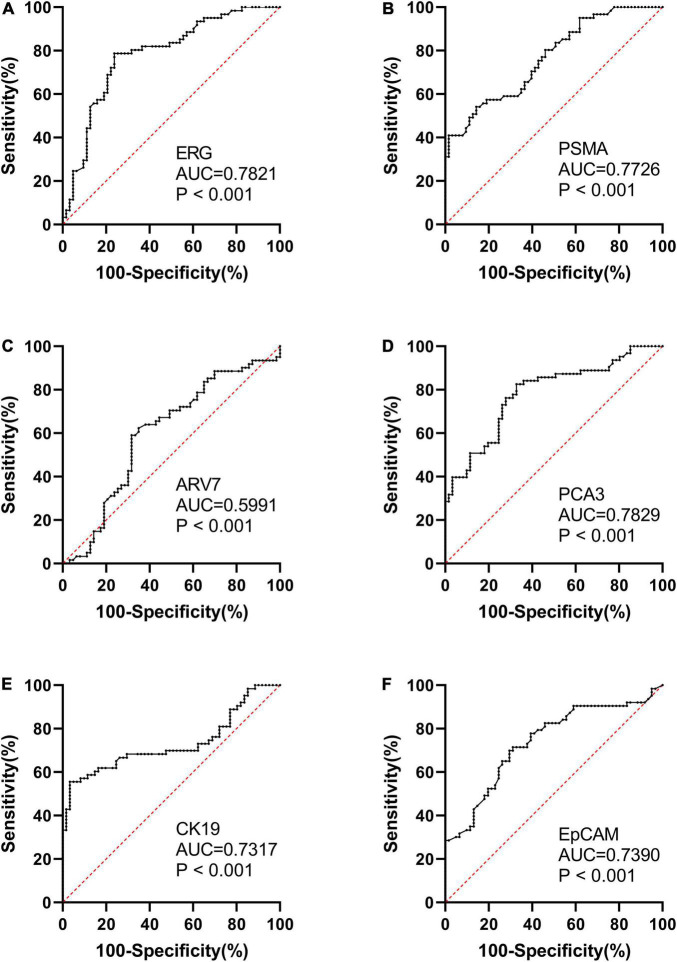
ROC curve analysis and AUC with 95% CIs of mRNAs analyzed in the urinary exosomes of PCa patients (*n* = 63) and healthy subjects groups (*n* = 61).

**FIGURE 3:**
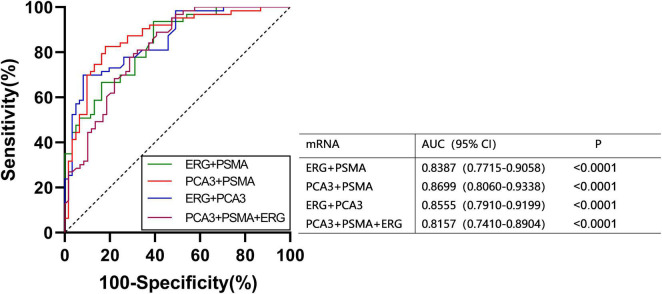
ROC curves were calculated for various combinations using logistic regression analysis.

We found that the expression of ERG, PSMA, PCA3, CK19, and EpCAM were significantly increased in patients with PCa (*P* < 0.001) (Figure 1). The RNAs were further analyzed by the ROC with 95% CIs. The AUC values of urinary exosomal ERG, PSMA, AR-V7, PCA3, CK19, and EpCAM were 0.7821 (95% CI,0.6999–0.8642), 0.7726 (95% CI,0.6854– 0.8478), 0.5591 (95% CI,0.4976–0.7007), 0.7829 (95% CI,0.7021–0.8633), 0.7317 (95% CI,0.6397–0.8240), and 0.7390 (95% CI,0.6512–0.8269), respectively (Figure 2). The result of ROC analysis demonstrated that the AUCs of three RNAs (ERG, PSMA, and PCA3) were over 0.75. Additionally, two of these candidate RNAs were combined together based on a logistic model, by which improved outcome was obtained, compared with individual RNA. Notably, the AUC of ERG + PSMA, PCA3 + PSMA, and ERG + PCA3 were over 0.85. The integration of a third candidate failed to achieve further improvement, when compared to the PCA3 plus PSMA model. The highest AUC of PCA3 plus PSMA was 0.8699 (Figure 3).

**FIGURE 4:**
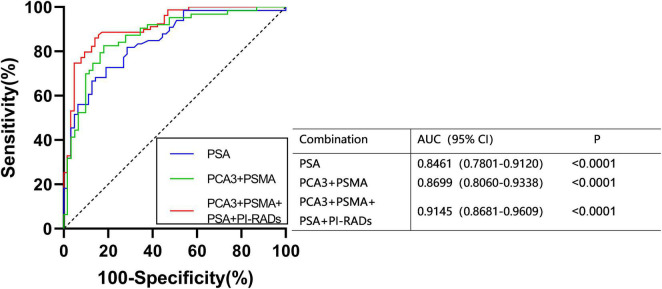
ROC curves were calculated for the combined PCA3, PSMA, PI-RADS, and PSA using logistic regression analysis.

PSA and PI-RADS are the most frequently applied diagnostic indicators of PCa. To figure out whether the combination of PCA3 and PSMA can serve as effectively as plasma PSA, the diagnostic efficacy of PCA3 + PSMA from urinary was evaluated and compared with PSA. In ROC analysis using a logistic model, PSA exhibited lower AUC values, compared to PCA3 + PSMA and PCA3 + PSMA + PSA + PI-RADS. Notably, PCA3 + PSMA + PSA + PI-RADS presented an AUC of 0.9145 (Figure 4).

**FIGURE 5:**
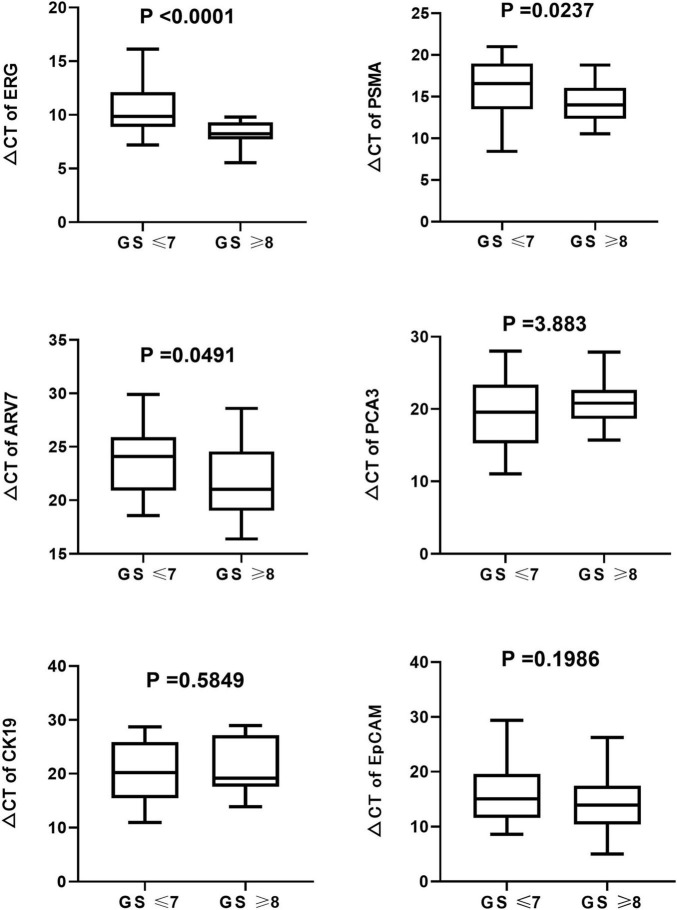
Boxplots of the six mRNAs between PCa patients (*n* = 63) and healthy subjects groups (*n* = 61) in urinary exosomes. Statistical test: U Mann–Whitney.

**FIGURE 6:**
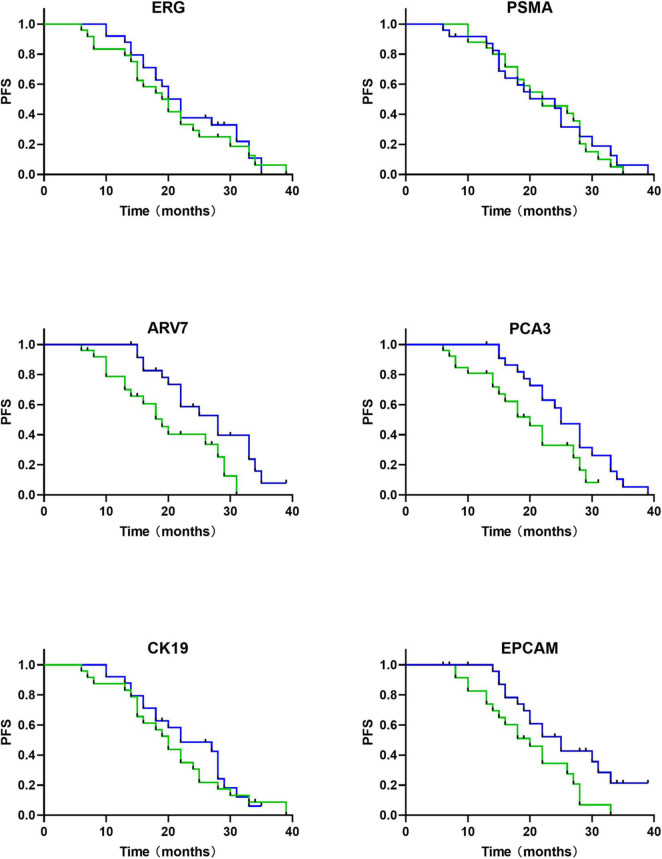
mRNAs based survival analysis in follow-up cohort of PCa patients (*n* = 63) (time in months). Kaplan-Meier curves show that relative expression levels of mRNA are associated with androgen-deprivation therapy (ADT) failure time (green, high expression of mRNA; blue, low expression of mRNA).

The expression of ERG, PSMA, and ARV7 were significantly (*P* < 0.05) different regarding varied Gleason score, where lower levels of ΔCt were detected in patients with higher Gleason score (Figure 5). However, no significant difference in the expression of PCA3, CK19 and EpCAM was observed among Gleason score groups. On the other hand, no significant difference in any RNA was found, compared with T stage. Kaplan-Meier curves depicted the association of PCA3, ARV7, and EpCAM in the time to failure of androgen deprivation therapy (ADT) which is defined as from the start of hormone-sensitive phase of ADT to the development of castration-resistant prostate cancer (Figure 6).

## Discussion

Previous studies have suggested that variable expression patterns of RNA in tissues have the potential to distinguish PCa from normal tissues. In terms of PCa biomarkers, urine, containing exfoliated PCa cells and other secretions, appears to be a challenging alternative of serum. In addition, the quantity of protein content in urine is proved to be lower than that in serum and plasma, thus the interference of isolating RNA-derived biomarkers can be reduced. The development of novel biomarkers for the diagnosis and prediction of PCa has been a major goal in the research on PCa, which can help improve diagnostic accuracy of PSA (17).

Extracellular vesicles, the membrane-bound vesicles, are secreted by cells in normal and pathological conditions, which can be found in all biological fluids, including blood, urine EVs, and saliva and categorized into different subtypes with respect to their subcellular origins. Exosomes, as a subtype of EVs, possess relatively small sizes (30–200 nm), and are released by multivesicular bodies (MVBs) pathway. Exosomes are rich in four-chain proteins, such as CD63, CD9, CD81, and other membrane-associated proteins including heat shock proteins (HSPs), integrins, lipid fixation proteins and glycoproteins, as well as various elements, such as TSG101 (18). It is well known that exosomes can deliver other structural and functional molecules, including metabolic enzymes, DNA, lipids, RNA, and mRNAs (19). Notably, several functions of exosomes have been proposed, such as cellular debris removal, cellular defense, immune regulation, and intercellular communication (20). Secreted exosomes can be taken up by recipient cells and impact their behavior (21). Several studies have shown that cargo uptake associated with exosomes can influence tumor formation, development, invasion, metastasis, and resistance (22, 23). Non-etheless, the exact functional role of urinary EVs in cancer remains unclear.

There are various methods to isolate exosomes. However, exosome studies have been limited by the traditional inconsistent isolation methods, nomenclature, and the lack of standardized protocols in data collection and analysis. Size-exclusion Chromatography (SEC) used in this study has been recommended as the most appropriate choice in separating exosomes from most proteins. The combined use of SEC and prostate cancer urinary exosomes has not been reported before. The SEC starting sample fluid was used as the mobile phase, and the porous gel filtration polymers containing cross-linked dextrose, agarose, polyacrylamide, or allyl dextran were applied as stationary phases (24). The stationary phases were set for different kinds of elution: first for larger particles, followed by smaller vesicles, finally against non-membrane bound proteins. Since larger particles cross fewer pores, compared to the smaller one, they cross shorter paths, and result in less time consumption in reaching the bottom of the column. SEC has been successfully used in isolation, purification and enrichment of exosomes from a series of biological fluids, for example plasma (25–27), serum (28–30), urine (31–34), cerebrospinal fluid (35, 36), synovial fluid (37), and nasal lavage (38). Excessive sample volumes and limited sample types are not required for the isolation, which are superior to other techniques. The results of the study reported at the ISEV 2020 conference have showed the superiority of SEC in isolating pure exosomes from human body fluids. This method was successfully applied in our study to isolate sufficient exosomes for following mRNA detection, and the rigor and reproducibility were ensured by the MISEV 2014/2018 guidelines.

In this study, we evaluated the value of the combination of ERG, PSMA, AR-V7, PCA3, CK19, and EpCAM in PCa diagnosis for the first time. The levels of these RNAs in urinary exosomes extracted from 63 PCa patients and 61 healthy controls were analyzed. We found that ERG, PSMA, PCA3, CK19, and EpCAM were significantly upregulated in urinary exosomes from PCa patients. A panel combining PCA3 and PSMA was considered as the best union to differentiate PCa patients and healthy individuals with an AUC of 0.870. The detection of the expression of PSA in serum and PI-RADS confirmed that the highest AUC of PCA3 + PSMA + PSA + PI-RADS was 0.914.

Until now, few studies have focused on combining the RNA of urine exosomes in PCa patients for prognosis. PCA3, discovered in 1999 with high expression levels in 53 out of 56 PCa specimens, is one of the most commonly used urine markers for the prognosis of PCa (13, 14). Real-time PCR were performed for PCA3 quantification (39), in which PCA3 was found to be detectable in prostate urine with a specificity of 90% in 16 out of 24 males with positive biopsy results (40). The knockdown of PCA3 has been previously proved to cause changes in expression levels of several transcript encoding AR cofactors, including the up-regulation of E-cadherin, claudin-3, and cytokeratin-18, and the down-regulation of vimentin (41). A meta-analysis reported (including 12,265 men who accepted PCA3 testing) a sensitivity and specificity of 65 and 73% in detecting prostate cancer, respectively, although the enrolled males with first or repeated biopsies were not separated (42). Tomlins et al. (21) have verified the fusions of the 5′ non-coding region of TMPRSS2 (transmembrane protease serine 2) with the ERG transcription factor gene (ETS-related gene) in prostate cancer tissues. Fusions between TMPRSS2 and ERG which were screened by PSA were reported to account for 90% of all ETS fusions. tMPRSS2: ERG rearrangement can tune the overexpression of ERG which is highly specific for PCa (22). The ExoDxProstate test measures ERG, PCA3, and SPDEF to determine the risk of GS7 or higher grade cancer at initial biopsy (AUC 0. 73–0.77) with the combination of SOC variables (9). According to our study, differences in PCA3 and ERG in urine-derived EVs between PCa and normal subjects were statistically significant. In addition, Kaplan-Meier curves depicted the relationship between PCA3 and time to failure of ADT.

Previous studies have proved the high expression of PSMA in advanced PC or mCRPC tissues (11). High PSMA expression in tumors is known to be related to higher tumor stage/Gleason score/preoperative PSA levels, up-regulated HER2 expression and increased biochemical recurrence risk (12). No correlation was found among PSMA mRNA levels in blood, tumor stage, Gleason score and serum PSA. Furthermore, the examination of PSMA mRNA in blood can predict the risk of biochemical recurrence after radical prostatectomy (43). Currently, urinary exosomal PSMA hasn’t been well applied in diagnosis of prostate cancer. In this study, our data showed that PSMA expression in urinary exosomes can distinguish PCa from healthy populations. Furthermore, combining PCA3 and PSMA expression in urinary exosomes showed an AUC of 0.870.

ARV7, an aberrantly spliced mRNA isoform of the androgen receptor, has been proved by increasing evidences that ARV7 mRNA abstracted from circulating tumor cells (CTCs) may be a prognostic marker which can both indicate the resistance against novel endocrine therapies, including abiraterone and enzalutamide (44) and play a role in the prediction of sensitivity toward chemotherapeutic, such as docetaxel and cabazitaxel (45, 46). Patient prognosis can be promoted with the utilization of the AR-V7 CTC test in treatment selection (47). A meta-analysis and systematic review demonstrated that CRPC exhibited a significantly higher proportion of AR-V7 positive, comparing with newly diagnosed PCa, where only 249 out of 950 patients with newly diagnosed PCa were AR-V7 positive (48). Similarly, our study also showed that ARV7 expression was not statistically significant in newly diagnosed PCa and normal subjects, but that ARV7 levels were associated with the time between the start of ADT at the hormone-sensitive stage and treatment failure.

The relationship between cytokeratin19 (CK19)/epithelial cell adhesion molecules (EpCAM) and PCa diagnosis have not been well investigated. Sunkara et al. (49) demonstrated a new method in which EVs were isolated from whole blood and plasma samples, then lysed and measured by ELISA, by which a significant difference was observed between PCa and healthy individuals in PSA, PSMA, and EpCAM with significantly different optical densities. Another study has identified that EpCAM expression level was significantly higher in PCa samples than that in benign and normal samples, which was verified by immunohistochemistry (15). One study has found that urinary CK19 mRNA can be used to assess (intraoperative) lymph node staging in PCa (50). In the present study, CK19 and EpCAM were also found to be of diagnostic value for primary prostate cancer. Moreover, we found a significant association of EpCAM with ADT failure time.

## Conclusion

In this study, SEC has been utilized as an effective and simple method to isolate exosomes, based on which PCa-related mRNAs in exosomes from urine were quantified and analyzed. The results demonstrated that the sensitivity and specificity in diagnosing PCa were promoted by the combination of PCA3 + PSMA. Furthermore, the diagnostic potential of ERG, PSMA, AR-V7, PCA3, CK19, and EpCAM in PCa were also evaluated, which illustrated that combining PCA3 + PSMA + PSA + PI-RADS gave the best diagnostic efficacy. With the upcoming enlargement of sample numbers and varieties which can be achieved through a future multicenter and larger cohort study, these new diagnostic indicators may play their significant roles in early diagnosis of PCa.

## Data Availability Statement

The raw data supporting the conclusions of this article will be made available by the authors, without undue reservation.

## Ethics Statement

The studies involving human participants were reviewed and approved by Medical Ethical Committee of Tongji Hospital of Huazhong University of Science and Technology. The patients/participants provided their written informed consent to participate in this study.

## Author Contributions

JG: writing – original draft and software. XZ: software and formal analysis. XW: supervision. YW: experiments. PL: review and editing. ZW: funding acquisition. CY: conceptualization, funding acquisition, and writing – review and editing. ZH: conceptualization, project administration, and resources. All authors contributed to the article and approved the submitted version.

## Conflict of Interest

YW was employed by the Wuhan YZY Medical Science and Technology Co., Ltd. The remaining authors declare that the research was conducted in the absence of any commercial or financial relationships that could be construed as a potential conflict of interest.

## Publisher’s Note

All claims expressed in this article are solely those of the authors and do not necessarily represent those of their affiliated organizations, or those of the publisher, the editors and the reviewers. Any product that may be evaluated in this article, or claim that may be made by its manufacturer, is not guaranteed or endorsed by the publisher.
